# Supplementary figures and images for: Diet and kwashiorkor: a prospective study from rural DR Congo
Source: PeerJ. 2014 Apr 15;2:e350. doi: 10.7717/peerj.350 (PMC3994641; doi:10.7717/peerj.350)

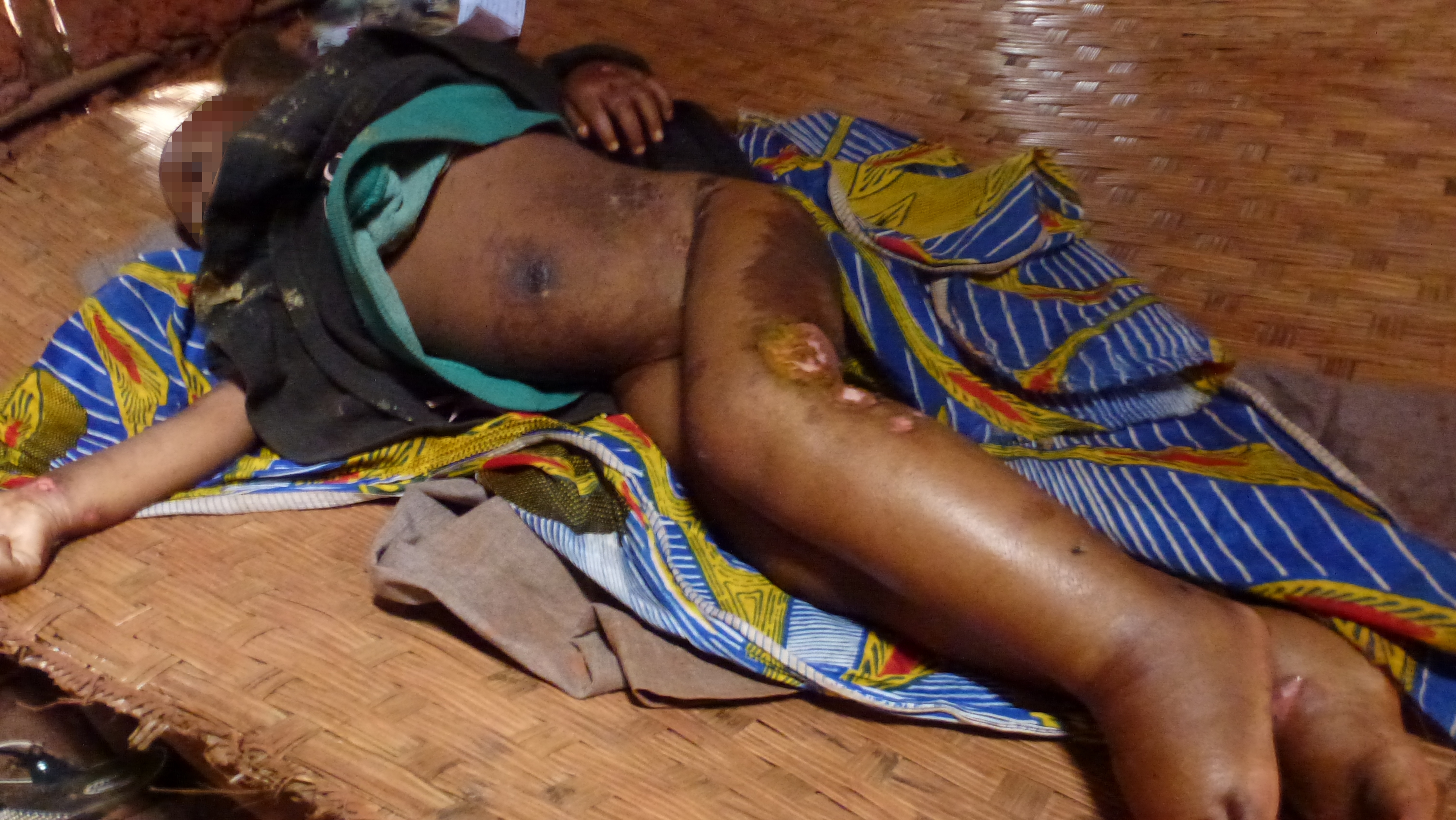

Supplement: Supplemental Information 1 — During a study in Bwamanda in 2013, this child was assessed according to nutritional status including occurance of oedema. The child on this photo was identified with kwashiorkor. [file peerj-02-350-s001.jpg]

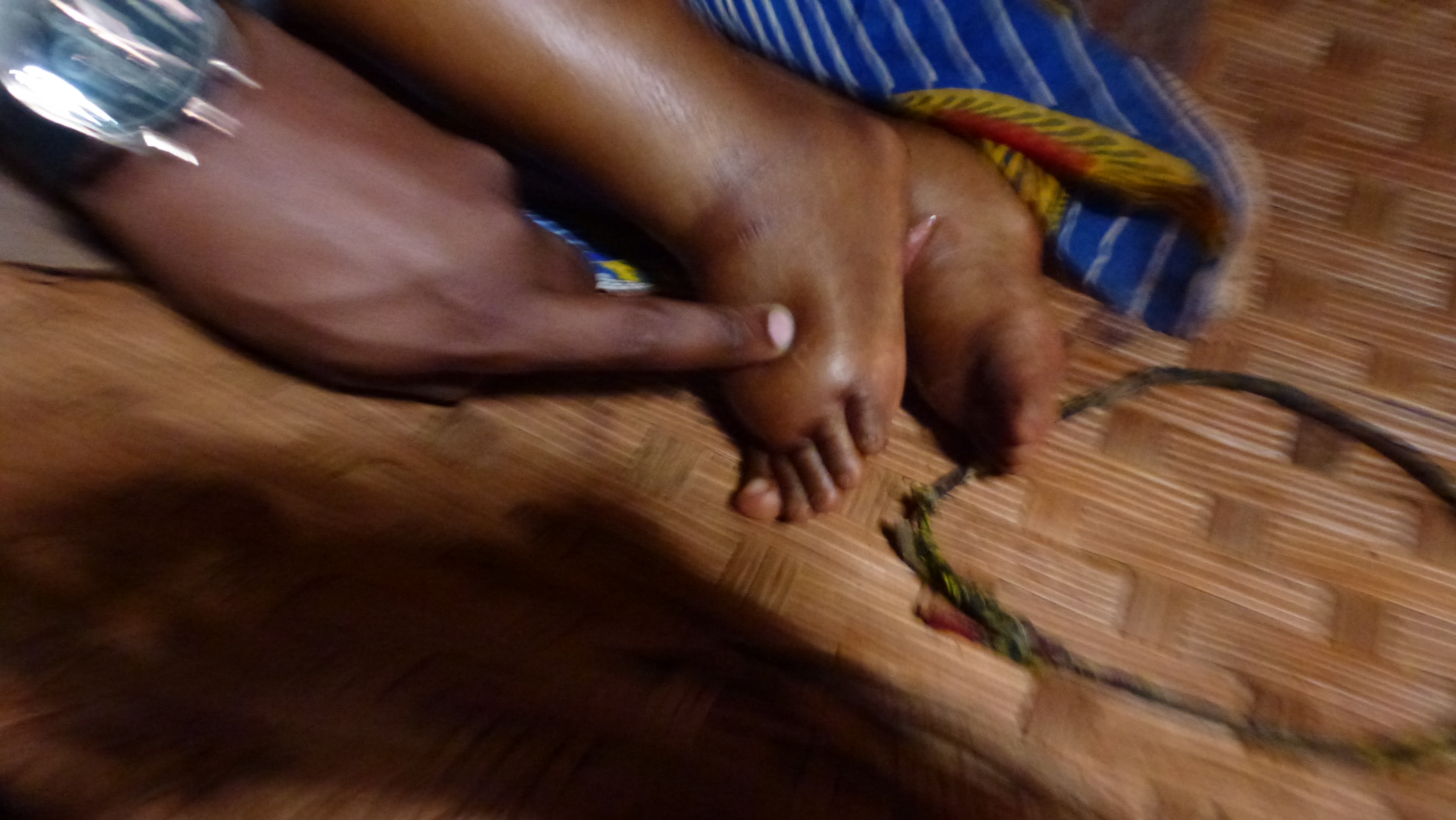

Supplement: Supplemental Information 2 — A Congolese doctor identifying pitting oedema on a child with kwashiorkor. [file peerj-02-350-s002.jpg]
